# Lung protective ventilation with lower tidal volumes and development of pulmonary complications in critically ill patients without ARDS

**DOI:** 10.1186/cc14334

**Published:** 2015-03-16

**Authors:** FD Simonis, A Serpa Neto, M Gama de Abreu, P Pelosi, MJ Schultz

**Affiliations:** 1 Academic Medical Center, Amsterdam, the Netherlands; 2 Hospital Isrealita Albert Einstein, São Paulo, Brazil; 3 University Hospital Carl Gustav Carus, Dresden, Germany; 4 IRCCS San Martino IST, Genoa, Italy

## Introduction

A large meta-analysis suggests that use of low tidal volumes benefits patients without ARDS [1] but most studies in this meta-analysis included patients receiving ventilation during general anesthesia for surgery. The aim of the present meta-analysis is to determine the association between tidal volume size and development of pulmonary complications in ICU patients.

## Methods

An individual patient data meta-analysis of studies of ventilation in ICU patients without ARDS. Corresponding authors of retrieved studies provided individual patient data. The primary outcome, pulmonary complications, was a composite of development of ARDS or pneumonia during hospital stay. Secondary outcomes included ICU and hospital length of stay, and in-hospital mortality. Patients were assigned to three groups based on tidal volume size (≤7 ml/kg predicted body weight (PBW), 7 to 10 ml/kg PBW, or ≥10 ml/ kg PBW).

## Results

Seven investigations (2,184 patients) were meta-analyzed. Pulmonary complications occurred in 23%, 28% and 31% respectively in the ≤7 ml/kg PBW, 7 to 10 ml/kg PBW and ≥10 ml/kg PBW group (adjusted RR, 0.72; 95% CI, 0.52 to 0.98; *P *= 0.042). Occurrence of pulmonary complications was associated with a lower number of ICU-free days and alive at day 28, a lower number of hospital-free days and alive at day 28 and increased in-hospital mortality.

## Conclusion

Ventilation with low tidal volumes is associated with a lower risk of development of pulmonary complications. Occurrence of pulmonary complications is associated with an increased ICU and hospital length of stay and in-hospital mortality in ICU patients without ARDS.
